# A Unique Presentation of an Intracranial Abscess Secondary to Retained Projectile after Debridement with Dural Closure

**DOI:** 10.7759/cureus.1328

**Published:** 2017-06-09

**Authors:** Jason Milton, Victor Awuor

**Affiliations:** 1 Neurosurgery, Ohio Health

**Keywords:** penetrating head trauma, abscess, projectile

## Abstract

Patients with penetrating head trauma with retained projectiles develop intracranial abscesses as a common complication. The most common presentation is a suddenly worsening headache. The most common pathogen identified is staphylococcus. Outcomes are related to adherence of Matson’s tenets.

This case study details the presentation of a 19-year-old patient that presented to the neurological surgery clinic without neurologic deficits. Further questioning revealed complaints of intermittent diffuse headaches with bilateral upper extremity shock-like sensation for two weeks. Eight weeks prior he had undergone right craniotomy, after a gunshot wound, for debridement and watertight dural closure. The patient denied symptoms of fever, chills, nausea, vomiting, diarrhea, or seizure. The patient presented with a noncontrast head computed tomography (CT) which revealed retained projectile fragments without clear evidence of abscess. On physical exam, the patient was without any neurological deficit. Laboratory investigation revealed normal white blood cell count, erythrocyte sedimentation rate, C-reactive protein, and negative blood cultures. Head CT with contrast revealed a large intracerebral abscess adjacent to the thalamus. The patient was taken to the operating room for repeat craniotomy with resection of the abscess and removal of the intracranial projectile fragments.

Post-operatively, the patient remained neurology intact. Intraoperative cultures were not significant for the growth of any bacteria. In eight weeks time, the patient returned to his employment and his baseline level of activity.

This case underscores the importance of thorough assessment in patients with retained intracranial projectiles as well as the need to routine follow-up. The unique presentation of this patient prompted further investigation which elucidated a lesion which correlated to his symptoms although laboratory assessment was without abnormality.

## Introduction

Approximately two million traumatic brain injuries occur annually in the United States at a cost of almost $25 billion. Fifty percent of all trauma-related deaths are attributed to traumatic brain injury with 35% of these injuries resulting from gunshot wounds to the head [1-2]. This accounts for approximately 20,000 injuries [3-4]. Patients with penetrating head trauma with retained projectiles develop intracranial abscesses as a common complication. The most common presentation is a suddenly worsening headache. The most common pathogen identified is *staphylococcus*. Advances made by Pasteur, Koch, and Lister with regard to bacteriology and asepsis have contributed to a reduction in the incidence of local and systemic infections as well as mortality [5].

Goals of surgical intervention include evacuation of mass lesions, debridement of necrotic tissue to prevent further swelling and ischemia, control of active hemorrhage, and debridement of necrotic tissue, bone, and foreign bodies [6]. Despite these relatively universal goals of surgery, patients that meet surgical indications can be difficult to identify. Outcomes for these patients are related to the fulfillment of Matson’s tenets (Table 1) [7]. The use of Grahm’s guidelines (Table 2) is helpful in identifying patients that are more likely to have positive outcomes if operative management is pursued. The guidelines place an emphasis on post-resuscitative Glasgow Coma Score (GCS) and initial CT (computed tomography) imaging [8].

**Table 1:** Matson's tenets. Dr Donald Matson outlined the purpose of far-forward neurosurgery in World War II. These principles hold true for neurosurgical management in the modern era.

Tenet	Current Application
I. Save life	Advanced trauma life support/advanced cardiac life support/far forward homeostasis and hemicraniectomy
II. Prevent infection	Watertight dural closure
III. Preserve nervous system function	Prevention of secondary neurologic injury through advanced neurocritical and neurointerventional care
IV. Restore anatomic function	Restore anatomic protection and contour

​

**Table 2:** Grahm's principles. The most commonly used criteria to determine operative management based on post-resuscitative GCS and CT imaging. GCS: Glasgow Coma Score; CT: Computed tomography.

Glasgow Coma Score	Surgery vs Nonoperative Management
3-5	Not associated with satisfactory outcome
5-7	Should be managed nonoperatively if their injury is multilobar, transventricular, or in the dominant hemisphere
>7	Should be considered for operative management

## Case presentation

**Figure 1:**
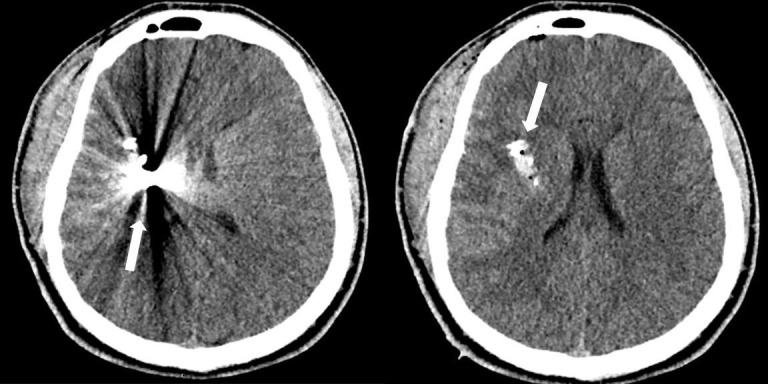
Presenting computed tomography (CT) head without contrast reveals penetrating head injury from high-velocity projectile with retained projectile adjacent to the right basal ganglia.

A 19-year-old male presented to our level 1 trauma center with a penetrating head injury from a high-velocity projectile of unknown caliber from a moderate distance. His neurologic exam was non-focal with a GCS of 15. Non-contrast CT of the head (Figure 1) revealed a gunshot wound of the right frontotemporoparietal region with a retained projectile in the region of the right basal ganglia. Due to the presence of an intact neurologic exam in this otherwise hemodynamically stable patient, the decision was made to pursue surgical intervention with goals of wound debridement and watertight dural closure. The perspective of the operating surgeon was that this patient was at high risk of developing infectious complications with potentially devastating neurologic consequences with non-operative management. The patient was started on broad-spectrum antibiotics, prophylactic anti-epileptic medication and taken to the operative theater within 12 hours of injury.

The patient was taken to the operating room for surgical debridement of the wound and watertight closure of the dura. The projectile was left in place due to it being adjacent to eloquent brain tissue which is in accordance with current standards of care with regard to surgical intervention in penetrating head trauma. He remained neurologically intact and hemodynamically stable throughout the peri-operative period. He was continued on antibiotics for 10 days post-operatively and on anti-epileptic therapy for seven days. The patient’s post-operative course was uneventful and he was discharged home on post-operative day 12 after thorough evaluation by physical therapy, occupational therapy, and physical medicine and rehabilitation.

**Figure 2:**
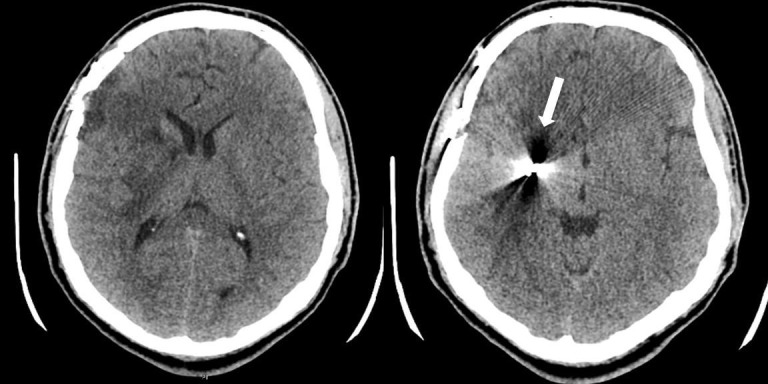
Post-operative computed tomography (CT) head without contrast revealing stable position of the retained high-velocity projectile adjacent to the right basal ganglia.

The patient was seen for a post-operative visit in two weeks and was without complaint. His neurologic exam remained stable and his surgical site appeared to be healing well with clean wound edges. He was instructed to follow up as needed. The patient was again seen for follow-up in the neurosurgery clinic two months post-operatively. He now complained of an intermittent shock-like sensation in his bilateral upper extremities that occurred multiple times daily since his previous follow-up. He presented to his clinic visit with a non-contrast CT of the head (Figure 2) that was read as having stable surgical changes from his recent post-operative imaging with no acute intracranial abnormality, but the retained foreign body was again noted.

**Figure 3:**
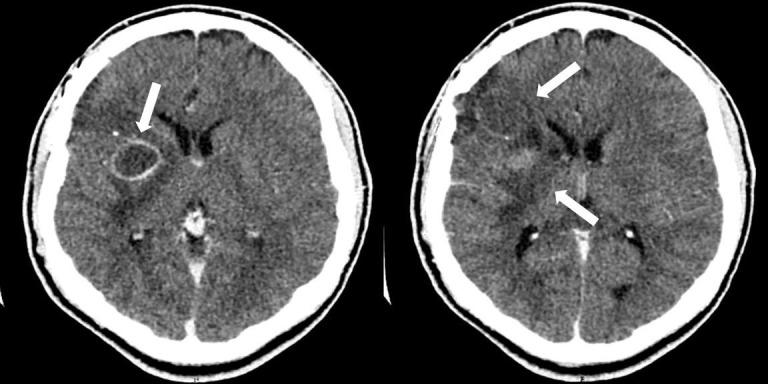
Post-operative computed tomography (CT) head with contrast performed at follow-up revealing two right frontal ring-enhancing lesions consistent with abscess.

**Figure 4:**
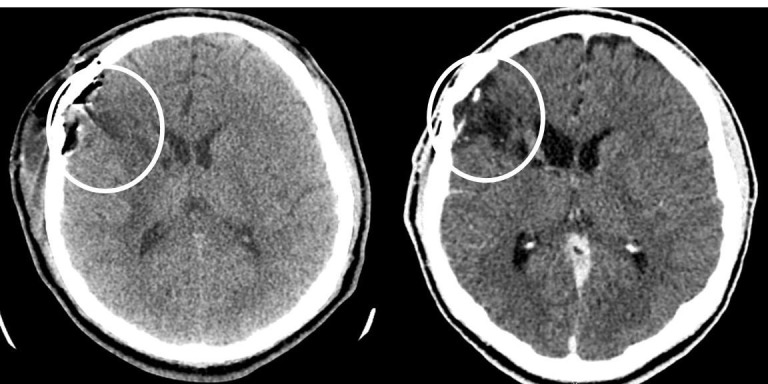
Post-operative computed tomography (CT) head without contrast revealing decompression of both previously noted lesions with evacuation of previously noted retained foreign body.

Due to the patient’s presentation, a contrast CT of the head (Figure 3), lead level, complete blood count, erythrocyte sedimentation rate, and C-reactive protein were ordered to further evaluate for possible abscess and metal toxicity. All labs were determined to be within normal limits. Contrast CT of the head revealed multiple ring-enhancing lesions of the right frontal lobe and basal ganglia adjacent to the previously noted foreign body. The patient returned to the operative theater for wound exploration, resection of abscesses, removal of the intracranial foreign body, and watertight dural closure. Post-operative CT confirms removal of the foreign body and abscess evacuation (Figure 4).

## Discussion

Delayed intracranial abscesses commonly present as a headache without distinguishing features, although usually poorly localized. Constitutional symptoms present in less than 50% of patients diagnosed with an intracranial abscess. Laboratory assessment is frequently normal in patients as it was in this case presentation. Delayed intracranial abscesses typically present at 2-3 months [9].

This unique presentation of an intracranial abscess in a neurologically intact patient highlights the importance of obtaining imaging with contrast during post-operative follow-up. The indescript presentation of headache and the multitude of complaints that may be related to an intracranial abscess may lead to an extensive workup that is unnecessary in the setting of a potential neurosurgical emergency.

## Conclusions

This unique presentation of an intracranial abscess in a neurologically intact patient highlights the importance of obtaining imaging with contrast during post-operative follow-up. The indescript presentation of headache and the multitude of complaints that may be related to an intracranial abscess may lead to an extensive workup that is unnecessary in the setting of a neurosurgical emergency. Thorough physical exam and proper imaging are necessary for adequate follow-up in these patients.
